# How Do We Study the Dynamic Structure of Unstructured Proteins: A Case Study on Nopp140 as an Example of a Large, Intrinsically Disordered Protein

**DOI:** 10.3390/ijms19020381

**Published:** 2018-01-27

**Authors:** Jung-Hyun Na, Won-Kyu Lee, Yeon Gyu Yu

**Affiliations:** 1 Department of Chemistry, Kookmin University 77, Jeongneung-Ro, Seongbuk-gu, Seoul 02707, Korea; protein@kookmin.ac.kr; 2 New Drug Development Center, Osong Medical Innovation Foundation, Osong Sengmyung-Ro 123, Osong-eup, Heungdeok-gu, Cheongju-si, Chungbuk 28160, Korea; gre7@kbiohealth.kr

**Keywords:** intrinsically disordered protein (IDP), nucleolar phosphoprotein 140 (Nopp140), conformational study, casein kinase 2 (CK2)

## Abstract

Intrinsically disordered proteins (IDPs) represent approximately 30% of the human genome and play key roles in cell proliferation and cellular signaling by modulating the function of target proteins via protein–protein interactions. In addition, IDPs are involved in various human disorders, such as cancer, neurodegenerative diseases, and amyloidosis. To understand the underlying molecular mechanism of IDPs, it is important to study their structural features during their interactions with target proteins. However, conventional biochemical and biophysical methods for analyzing proteins, such as X-ray crystallography, have difficulty in characterizing the features of IDPs because they lack an ordered three-dimensional structure. Here, we present biochemical and biophysical studies on nucleolar phosphoprotein 140 (Nopp140), which mostly consists of disordered regions, during its interaction with casein kinase 2 (CK2), which plays a central role in cell growth. Surface plasmon resonance and electron paramagnetic resonance studies were performed to characterize the interaction between Nopp140 and CK2. A single-molecule fluorescence resonance energy transfer study revealed conformational change in Nopp140 during its interaction with CK2. These studies on Nopp140 can provide a good model system for understanding the molecular function of IDPs.

## 1. Introduction

**Figure 1:**
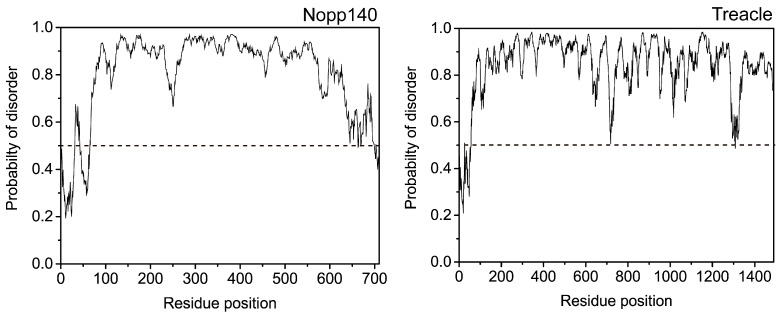
Computational prediction of disordered regions in nucleolar phosphoprotein 140 (Nopp140) and Treacle using IUPred server (http://iupred.enzim.hu/) [9]. If the residue value of these proteins exceeds a threshold (dotted line), the residue is considered disordered.

A well-defined tertiary structure was considered until recently to be the determining factor for biological and biochemical function of proteins. Active sites of enzymes or interaction domains of signaling proteins usually consist of amino acid residues in their tertiary structure. However, stretches of amino acids lacking a stable tertiary structure are often found in linker regions between well-folded domains. These flexible linker regions provide relative motional freedom between the connected domains, as observed in the linker region between the Fab and Fc domains of immunoglobulin G. On the other hand, long stretches of unstructured regions (usually >40 amino acids) are frequently found in various proteins, and these disordered regions are considered to be an intrinsic property of the protein. Proteins containing such disordered regions are called intrinsically disordered proteins (IDPs) or intrinsically unstructured proteins [1]. Genome-wide analysis suggests that approximately 25% of the proteins in eukaryotes contain disordered regions with >50 amino acids [2]. Even in archaea and bacteria, a significant portion of proteins contain disordered regions [2]. In humans, IDPs are found in various protein families, such as transcription factors, amyloid proteins, ribosomal proteins, and ribonucleoprotein complexes. Among them, IDPs, such as nucleolar phosphoprotein 140 (Nopp140) and Treacle [3], are distinctive IDPs which consist of >80% intrinsically disordered regions (IDRs) (Figure 1). The length of IDRs in these proteins is often >200–300 aa, and these sequences are characterized as having a high percentage of disorder-promoting residues [4] (Table 1). Although these IDPs lack a well-folded domain structure, they have several motifs that can serve as interaction sites with other biomolecules, such as DNA, RNA, or protein [5]. Previous studies have indicated that these interactions are associated with numerous biological functions and human disorders [6,7,8]. During interactions with target proteins, IDPs, particularly IDRs, may undergo conformational changes. It is challenging to characterize structural features of IDPs during interaction because there are only a limited number of experimental techniques which are useful in the structural analysis of IDPs. In this review, we summarize recent experimental methods targeting IDPs, particularly those which mainly consist of IDRs, and further present Nopp140 as an example for structural analysis using experimental methods, such as single-molecule fluorescence resonance energy transfer (smFRET), electron paramagnetic resonance (EPR), nuclear magnetic resonance (NMR), and circular dichroism (CD).

## 2. Molecular Mechanisms of IDPs: Regulation of the Target Protein by Specific Interaction

Most IDPs execute their function via molecular recognition of target molecules, except for a few IDPs that act via entropic function [10]. Target molecules of IDPs are nucleic acids, proteins, or small molecules. The function of the target molecules is altered after interaction with IDPs. On binding, IDPs can alter the activity of the target molecule in numerous ways. One well-characterized example is p53. IDR in the N-terminus of p53 (TAD of p53) interacts with p53-specific E3 ubiquitin ligase Mdm2. Upon binding, Mdm2 continuously ubiquitinates p53 and mediates its degradation by proteasomes [11]. In the p53 TAD, a 15-aa peptide serves as the binding site for the hydrophobic pocket at the N-terminus of Mdm2. Structural analysis of the interaction between Mdm2 and the binding peptide of p53 indicates that the binding peptide of p53 adopts a helical structure from a more disordered conformation [12]. IDPs often interact with several subunits in large multi-subunit complexes, such as ribonucleoprotein particles (RNPs), ribosomes, the cytoskeleton, or transcription pre-initiation complexes. In this case, IDPs serve as assemblers. U1-70k is IDP in which the disordered regions represent 60% of the whole protein. Structural analysis of U1 snRNP complexes showed that U1-70k wraps around the core domain of U1 snRNP and interacts with several components of the U1 snRNP particle [13]. This configuration implies that IDPs can stabilize the interaction between subunits in multi-subunit complexes. The structure of this complex demonstrates how IDP can interact with several protein components, stabilizing the complex protein structure as well as its own disordered regions.

## 3. Conformational Study of IDPs Using Experimental Techniques

Because IDPs are associated with various biological functions related to human disorders [6,7,8], it is important to understand their physiological properties. A conformational study is a common approach for understanding underlying mechanisms of protein function. Compared with globular proteins, it is difficult to study structural properties of IDPs using conventional methods, such as X-ray crystallography, because of the inherent structural flexibility of IDPs. For these reasons, bioinformatical approaches, such as using amino acid sequences to deduce the properties of IDPs, are preferred for their identification and structural characterization [5]. Bioinformatical analysis can predict disordered regions and specific regions related to protein–protein interaction [5]. For detailed characterization of properties, such as structural dynamics in solution, however, a combination of bioinformatical and experimental methods are required [14]. Here, we introduce some of the preferred experimental methods for understanding structural features of IDPs.

### 3.1. NMR Spectroscopy

NMR spectroscopy is a structural biology technique which is suitable as a complement to X-ray crystallography. A triple-resonance experiment (^13^C, ^15^N, and ^1^H), which detects sequential connectivity between neighboring residues, is typically used. For this experiment, it is necessary to purify isotope-labeled proteins (with ^13^C and/or ^15^N) expressed in culture media containing ^13^C glucose, ^13^C acetate, and/or ^15^NH_4_Cl. The NMR signal of protein sometimes display severe spectral overlap [15]. To overcome this problem, enhanced NMR technique and spectral signal analysis by sparse multi-dimensional Fourier Transform processing are used [15].

NMR may be suited for the conformational characterization of IDPs. The conformation of IDPs is sensitive to environmental changes (e.g., pH, temperature, ionic strength, and presence of ligands and/or binding molecules). Environment-induced conformational changes in IDPs can be detected by an NMR chemical shift [15,16]. This chemical shift, a sophisticated reporter of the backbone conformation, also indicates protein dynamics [17] and binding sites of ligands and/or other proteins. Compared with NMR of globular proteins, that of IDPs presents narrow line shapes [15,16,18,19,20,21] due to the highly dynamic nature of the polypeptide chain. Thanks to these narrow line shapes, the signal-to-noise ratio (S/N ratio) in NMR spectra of IDPs is usually high. However, S/N ratio can be low under physiological conditions (pH close to 7.0 and temperature about 30 °C) due to the exchange of amide protons with the solvent. This limit can be mostly solved by using direct ^13^C detection [22].

### 3.2. EPR Spectroscopy

EPR spectroscopy is useful for studying the structure and dynamics of macromolecules. Site-directed spin labeling (SDSL) EPR was developed by W. L Hubbel, and it is useful in characterizing protein structure and dynamics because most proteins do not have a paramagnetic center [23,24]. Nitroxides, which are stable free radicals, have an unpaired electron which is detectable by EPR. The EPR spectra of a nitroxide-labeled protein can provide information about: (i) the chain mobility at the label position; (ii) distance to other paramagnetic centers (using the double electron–electron resonance method); and (iii) accessibility of the solvent and oxygen [25].

Cysteine substitution mutagenesis of the protein, i.e., removing unwanted inherent cysteine (cysteine to serine) or introducing cysteines by site-directed mutagenesis, is the most common spin-labeling strategy [25]. The introduced thiol group is modified by spin-labeling agents such as (1-oxyl-2,2,5,5-tetramethyl-pyrroline-3-methyl)-methanethiosulfonate (MTSSL). Although MTSSL is small and has a minor impact on the protein structure [26], it is important to confirm differences in the structure and function of the spin-labeled protein compared with that of the wild-type. Several IDRs of IDPs were studied using the SDSL EPR technique [25,27,28,29,30,31,32,33,34]. The EPR spectra of IDRs showed sharp peaks due to the fast motion of the EPR probe, which results from the IDRs’ inherent flexibility.

### 3.3. CD Spectroscopy

CD spectroscopy in the far ultraviolet region (175–250 nm) is a particularly useful and simple method for characterizing the secondary structure (alpha helix, beta structure, polyprolin type II (PPII) helix, and random coil) [35] of proteins because of the small amount of sample required, user-friendly method, and the possibility for sample recovery. Generally, CD spectra of IDPs present a strong negative band near 200 nm [18,21,36,37,38], similar to those of random coils, supporting the theory that IDPs lack well-defined secondary structures. CD spectra of IDRs dramatically change when a change in the secondary structure is induced because of environmental factors [39]. Because CD spectra of alpha helices and beta sheets have distinct properties (alpha helix: two minima at 208 and 222 nm and one maximum at 190 nm; beta sheet: one maximum at 198 nm and one minimum at approximately 217 nm), CD spectra provide quantitative structural data for IDPs. Although the distinct signature of PPII in CD spectrum, a positive peak at 217 nm, is sometimes obscured by negative contribution from alpha helices and beta sheets, the presence of PPII conformation in IDPs is shown by CD analysis [40].

### 3.4. Single-Molecule Fluorescence Resonance Energy Transfer

As described above, NMR, EPR, and CD spectroscopy can provide valuable information about structural features and/or dynamics of IDPs. However, these methods cannot detect the dynamic heterogeneity of each individual molecule because of ensemble and time averaging of the signal [15,41]. Single-molecule fluorescence techniques are good alternatives to overcome the limitations of the former methods. smFRET experiments, one of the single-molecule fluorescence methods, is useful for studying structural features of IDPs [27,41,42,43,44,45,46,47]. smFRET is based on the transfer of excited-state energy from the donor to acceptor fluorescence dyes. smFRET provides information relating to conformation and conformational transitions induced by environmental changes both in the individual molecule and at the subunit level [41]. smFRET also measures intra- or intermolecular distance between the donor and acceptor fluorophores. smFRET analysis requires two or more fluorescent dye labels at different positions on the protein. The most common approach for fluorescence labeling is cysteine substitution mutagenesis, as described above in the EPR experiment. Maleimide-conjugated chromophores can attach to cysteine in specific regions. Cyanine (Cy) [48] or Alexa Fluor series [49] are suitable dyes for smFRET because of their high fluorescence quantum yield, small size, and stability. Recently, cysteine labeling combined with methods that involve non-natural amino acids have been used for studying multiple regions using three-color FRET [47].

## 4. Nopp140 as a Novel Class of IDP

**Figure 2:**
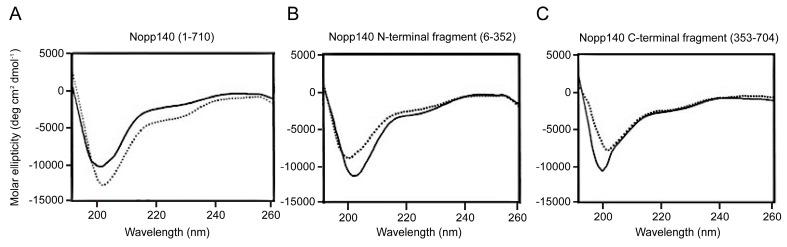
CD spectra of: (**A**) Nopp140; (**B**) the N-terminal (residues 6–352); and (**C**) C-terminal fragments (residues 353–704) of Nopp140. Phosphorylated and unphosphorylated forms are represented as solid and dotted lines, respectively (experimental data taken from [18], with permission from ©2012 KCS Publications).

Nopp140 is a nucleolar protein and shuttles between the nucleolus and cytoplasm in mammalian cells [50]. It functions in nucleolus formation during cell division [51] and might be involved in the assembly of pre-ribosomal subunits [50]. It interacts with proteins essential for cellular function, such as RNA polymerase I [52], NAP57 [53], snRNPs [52], p80 coilin [50], and casein kinase 2 (CK2) [54]. Nopp140 contains 710 amino acids, and >80% of its structure is deemed to be disordered based on several disordered region prediction algorithms [27,55]. This prediction result together with CD spectra (Figure 2) and high protease sensitivity of Nopp140 [55] shows that Nopp140 is IDP.

### 4.1. Interaction Between Nopp140 and CK2

CK2 is a ubiquitous serine/threonine kinase that phosphorylates various proteins related to crucial cellular functions, such as cell division, proliferation, and signal transduction [56,57,58,59]. A high level of CK2 expression has been observed in many cancers [60], suggesting that CK2 is a potential anti-cancer drug target.

Nopp140 has approximately 80 target sequences that can be phosphorylated by CK2 [61]. Nopp140 can bind to both CK2 (catalytic and regulatory) subunits, and the binding affinity of the phosphorylated Nopp140 is higher than that of unphosphorylated Nopp140 [62,63]. Nopp140, particularly in its phosphorylated form, can also regulate the catalytic activity of CK2 [62]. These results suggest that Nopp140 is a CK2 substrate and is a negative regulator of CK2. A yeast two-hybrid study using Nopp140 fragments showed that the C-terminus fragment (residues 528–704) binds regions of the catalytic subunit (CK2α) [63].

### 4.2. Biophysical Study of Nopp140

**Figure 3:**
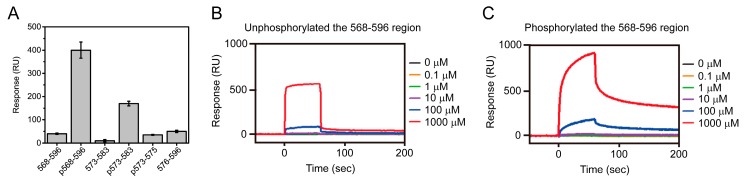
SPR analysis of the interaction between catalytic subunit of CK2 (CK2α) and Nopp140 fragments. “p” indicates the phosphorylation of Ser574 in Nopp140 fragments. (**A**) Binding affinities of Nopp140 fragments with CK2α. SPR sensorgrams of: (**B**) Nopp140 (568–596); and (**C**) the phosphorylation of Ser574 in Nopp140 (568–596) with CK2α. These sensorgrams were obtained for 0.1 (orange), 1 (green), 10 (purple), 100 (blue), and 1000 μM (red) of Nopp140 fragments, respectively (experimental data taken from [64], with permission from ©2013 National Academy of Sciences).

To understand the CK2-regulating mechanism and conformational change of Nopp140 during its interaction with CK2, we prepared a variety of Nopp140 fragments based on the C-terminus fragment of Nopp140. Surface plasmon resonance (SPR) experiments using the Nopp140 fragments indicate that residues 568–596 and the phosphorylation of Ser574 in Nopp140 fragments are crucial for interaction with CK2α (Figure 3) [64]. The interaction between the 568–596 region and CK2α is interrupted by D-*myo*-inositol 1,2,3,4,5,6-hexakisphosphate (IP_6_) [64], an important regulator of many biological functions. These results together with the complex model structure of the 568–596 region and the CK2α [27] suggests that the phosphorylated Ser574 and IP_6_ share the same binding site for CK2 [64], which is the key site for regulation of CK2 activity by Nopp140.

**Figure 4:**
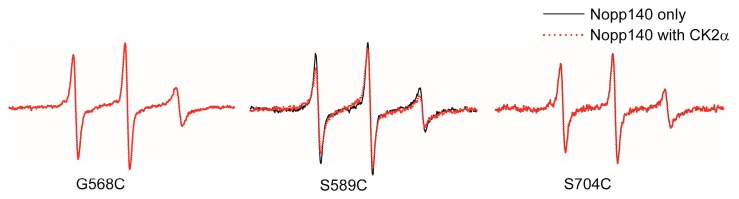
EPR spectra of spin-labeled C-terminus Nopp140 fragments. The EPR spectra of Nopp140 fragments in the absence and the presence of CK2α are represented are shown as solid (black) and dotted-lines (red), respectively (experimental data taken from [27], with permission from ©2016 Elsevier).

EPR spectra created using the spin-labeled (MTSSL) Nopp140 fragments showed sharp peaks because of the fast motion of the nitroxide group in MTSSL (Figure 4) [27]. Among the MTSSL-labeled Nopp140s, only the EPR spectrum of the 589 fragments (those spin-labeled at residue 589) was changed to a broader line shape in the presence of CK2α (Figure 4) [27]. These results imply that MTSSL-labeled Nopp140s were labeled in disordered regions and the region near amino acid 589 might be crucial for binding to CK2.

**Figure 5:**
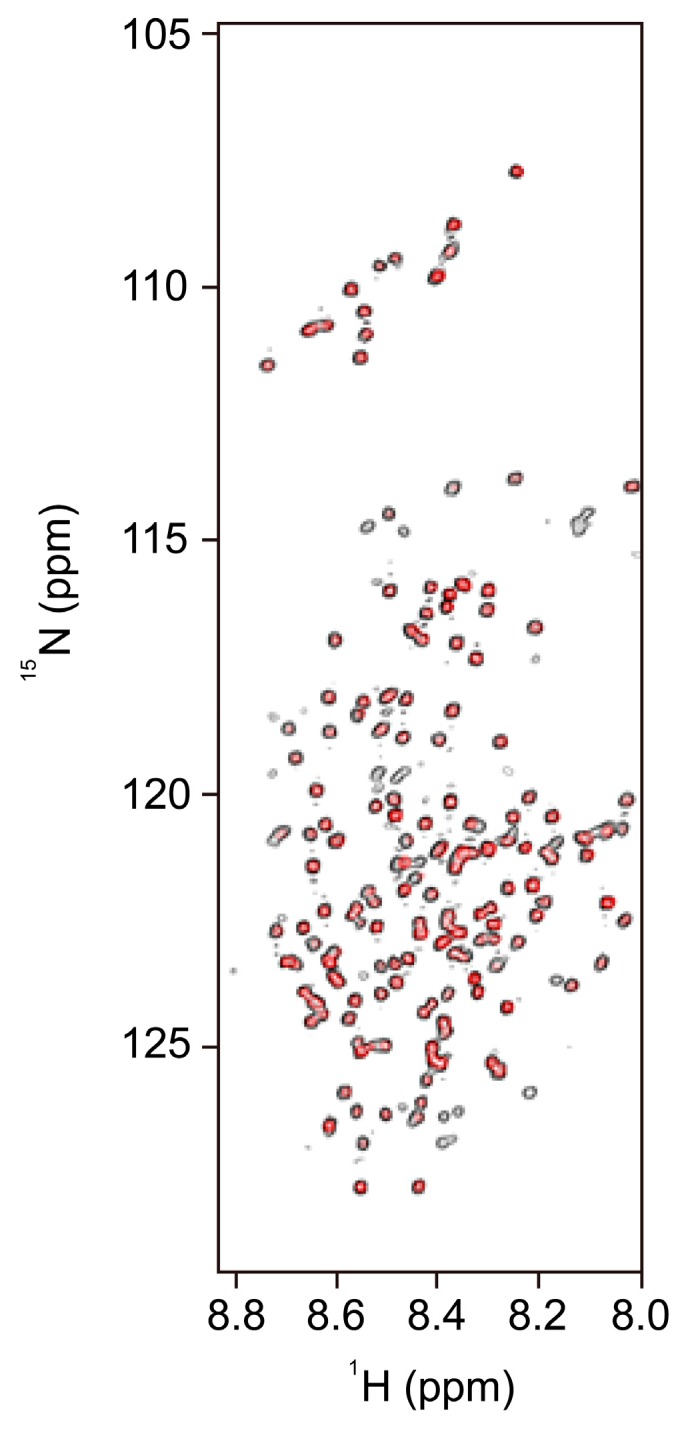
^1^H-^15^N spectra of the C-terminus Nopp140 fragment (residues 528–704, black) and with mitoxantrone (red) (experimental data taken from [18], with permission from ©2012 KCS Publications).

2D ^1^H-^15^N heteronuclear single quantum coherence spectroscopy (HSQC) NMR spectra of the C-terminus fragment presented a narrow line shape (Figure 5), typical of the ^1^H-^15^N HSQC NMR spectra of IDPs [18], which is another result that supports the theory that the C-terminus of Nopp140 is a disordered region. NMR spectra of Nopp140 are shifted when C-terminus fragments are mixed with mitoxantrone (Figure 5), an anti-cancer agent, demonstrating that Nopp140 interacts with mitoxantrone [18]. An in vitro kinase assay of CK2 showed that the reduced inhibitory activity of Nopp140 against CK2 by IP_6_ was recovered by mitoxantrone [18]. Results of the kinase assay, together with the ^1^H-^15^N HSQC NMR experiment of Nopp140 in the presence of mitoxantrone, imply that mitoxantrone stabilizes the regulation of CK2 by Nopp140 by enhancing the interaction between Nopp140 and CK2 [18].

**Figure 6:**
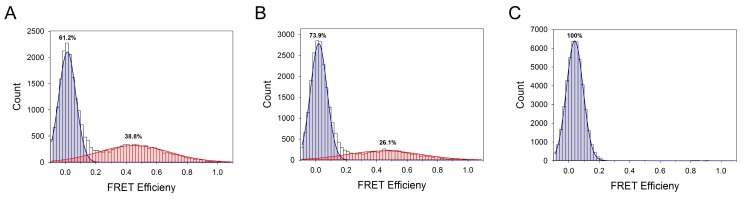
smFRET histograms of double-labeled: (**A**) Nopp140-352/589 only; (**B**) with CK2α; and (**C**) in 6 M guanidine hydrochloride. Two Gaussian peaks distributions having a FRET-peak centered at 0.03 and 0.45 E are shown as blue and red, respectively (experimental data taken from [27], with permission from ©2016 Elsevier).

Double fluorescence-labeled (Cy3 and Cy5 probes) Nopp140 fragments were used for FRET experiments [27]. An ensemble FRET experiment showed that the FRET intensity of the Nopp140-352/589 fragment was higher than that of other fragments (in order, from high to low: Nopp140-352/589 > Nopp140-352/467 > Nopp140-352/660 > Nopp140-352/704) [27], indicating that the estimated distances between the fluorescence labels in solution are not proportional to the number of amino acids. This result implies that the structure of Nopp140 is not linear or in a locally constrained conformation. The Nopp140-352/589 intensity substantially decreased in the presence of CK2α, which suggests that conformational distribution or the structure of the region near amino acid 589 is changed when CK2α binds to the C-terminus of Nopp140 [27]. Single-molecule FRET experiments using double-labeled Nopp140-352/589 fragments provided more detailed information (Figure 6). The FRET histogram of Nopp140-352/589 showed two Gaussian distributions (the low- and middle-FRET population) (Figure 6A) [27]. Because the FRET histogram in the presence of 6 M guanidine hydrochloride presented only one Gaussian distribution (Figure 6C) [27], the C-terminus fragment of Nopp140 has at least two structural conformations in its isolated form. Consistent with the ensemble FRET results, approximately 30% of the middle-FRET population shifted to low FRET in the presence of CK2α (Figure 6B) [27]. This population change indicated that the structure of Nopp140 became more restricted in its conformation because of its interaction with CK2α.

## 5. Conclusions

IDPs harboring a defined disordered region or IDPs mostly comprising disordered regions are commonly observed in most organisms, and their significance in various biological phenomena is clear. In particular, IDPs in which the disordered regions cover >60–80% of the whole protein are of interest with respect to their mechanism of action. These IDPs possess several motifs which can serve as interaction sites for different binding proteins, and the activity of a binding protein can be modified upon interaction with IDPs. Nopp140 is a good example of such IDP. It lacks any well-folded domains in its entire sequence of 710 aa, and it can interact with various proteins or RNAs, such as CK2α, RNA polymerase I, NAP57, p80 coilin, and snRNPs. The modulation of the kinase activity of CK2 by Nopp140 has been studied in detail. CK2-dependent phosphorylation of Nopp140 and inhibition of the kinase activity of CK2 by phosphorylated Nopp140 indicates that Nopp140 plays the role of a negative regulator to CK2 [62,64]. Further biophysical studies on Nopp140 during its interaction with CK2 by smFRET, EPR, and NMR revealed increased rigid conformation of Nopp140 at or near the CK2 binding site [27]. It could be said that the interacting molecules of Nopp140 are specifically positioned. A comprehensive study of the dynamic interaction between proteins (or RNAs) mediated by mostly disordered proteins will reveal much that is waiting to be uncovered.

## Figures and Tables

**Table 1:** The percent of disordered-promoting residues (P, E, S, Q, and K) of Nopp140 and Treacle.

Disordered protein	Nopp140	Treacle
% of disordered-promoting residues	54.4	49.1

Nucleolar phosphoprotein 140 (Nopp140).
